# Subcollicular projections to the auditory thalamus and collateral projections to the inferior colliculus

**DOI:** 10.3389/fnana.2014.00070

**Published:** 2014-07-18

**Authors:** Brett R. Schofield, Jeffrey G. Mellott, Susan D. Motts

**Affiliations:** ^1^Auditory Neuroscience Group, Department of Anatomy and Neurobiology, Northeast Ohio Medical UniversityRootstown, OH, USA; ^2^Department of Physical Therapy, Arkansas State UniversityJonesboro, AR, USA

**Keywords:** medial geniculate nucleus, lateral lemniscus, superior olive, parallel pathways, binaural, brain evolution, paralemniscal area, reticular formation

## Abstract

Experiments in several species have identified direct projections to the medial geniculate nucleus (MG) from cells in subcollicular auditory nuclei. Moreover, many cochlear nucleus cells that project to the MG send collateral projections to the inferior colliculus (IC) (Schofield et al., 2014). We conducted three experiments to characterize projections to the MG from the superior olivary and the lateral lemniscal regions in guinea pigs. For experiment 1, we made large injections of retrograde tracer into the MG. Labeled cells were most numerous in the superior paraolivary nucleus, ventral nucleus of the trapezoid body, lateral superior olivary nucleus, ventral nucleus of the lateral lemniscus, ventrolateral tegmental nucleus, paralemniscal region and sagulum. Additional sources include other periolivary nuclei and the medial superior olivary nucleus. The projections are bilateral with an ipsilateral dominance (66%). For experiment 2, we injected tracer into individual MG subdivisions. The results show that the subcollicular projections terminate primarily in the medial MG, with the dorsal MG a secondary target. The variety of projecting nuclei suggest a range of functions, including monaural and binaural aspects of hearing. These direct projections could provide the thalamus with some of the earliest (i.e., fastest) information regarding acoustic stimuli. For experiment 3, we made large injections of different retrograde tracers into one MG and the homolateral IC to identify cells that project to both targets. Such cells were numerous and distributed across many of the nuclei listed above, mostly ipsilateral to the injections. The prominence of the collateral projections suggests that the same information is delivered to both the IC and the MG, or perhaps that a common signal is being delivered as a preparatory indicator or temporal reference point. The results are discussed from functional and evolutionary perspectives.

## Introduction

The general view of the ascending auditory pathways includes divergent projections from the cochlear nucleus to multiple brainstem nuclei and then a re-convergence of projections from most of these nuclei to the inferior colliculus (IC). The IC then provides the ascending input to the medial geniculate nucleus (MG), the main auditory center in the thalamus and source of projections to auditory cortex. The projections from the IC to the MG travel in the brachium of the IC, so one might predict that cutting this fiber bundle bilaterally would eliminate all functional hearing. However, bilateral lesions of the brachia do not eliminate sensory input to the auditory cortex or prevent various auditory behaviors.

Galambos et al. (1961) showed that bilateral section of the brachium had little effect on click-evoked activity in the auditory cortex of cats. A series of behavioral studies in cats with similar lesions showed clearly that the animals could localize and orient to a sound source and also learn frequency discriminations (Goldberg and Neff, 1961; Jane et al., 1965; Casseday and Neff, 1975). The animals' performance in these tasks was much degraded compared to unlesioned animals, and they learned discriminations only with difficulty. However, these behavioral results demonstrate clear auditory function in the absence of IC projections to the MG. These functions were ascribed to an “extralemniscal” pathway that bypasses the IC. Our understanding of this pathway, and in particular its origins, has been progressing slowly for several decades.

Morest (1965) described a lateral tegmental pathway that terminates in the dorsal MG. Interestingly, he described the course of these fibers as ventral to the tectum and medial to the lateral lemniscus and the brachium of the IC. As such, these fibers can be spared when the brachium of the IC is lesioned, leaving the lateral tegmental fibers as the presumptive basis for auditory function that remained after the lesions described above. The pathway was believed to originate in several areas of dorsal midbrain tegmentum, including the sagulum and the cuneiform nucleus as well as more rostral areas. Henkel (1983) and Casseday et al. (1989) described projections to the MG from cells in or near the ventral nucleus of the lateral lemniscus (VLL). Angelucci et al. (1998) subsequently described subcollicular projections to the MG that originated from the dorsal tegmental regions and areas near the VLL, as described in earlier studies, and also from additional cells in medial and lateral superior olivary nuclei. Finally, more recent work has revealed a direct projection to the MG from the cochlear nucleus (Malmierca et al., 2002; Anderson et al., 2006; Schofield et al., 2014). This projection terminates primarily in the medial subdivision of the MG (MGm) and presumably underlies the short-latency auditory responses from single MGm neurons (Anderson et al., 2006; Anderson and Linden, 2011). Anderson and Linden (2011) suggest that this pathway can carry narrowly tuned information about acoustic stimuli. Furthermore, the MGm provides widespread projections to the auditory cortex as well as to the amygdala, where stimuli can be assessed for salience and provide important guidance for subsequent behavior. Thus, the pathway that bypasses the IC to reach the thalamus could have very widespread effects in the forebrain.

We recently described the cells in the cochlear nuclei that project to the MG in guinea pigs (Schofield et al., 2014). These projections arise from a small number of cells in the deep layer of the dorsal cochlear nucleus and a greater number of cells in the ventral cochlear nucleus. Furthermore, a substantial proportion of the cochlear nucleus cells that project to the MG have collateral projections to the IC. This implies that the same fast signal sent by the cochlear nucleus to the MG is also sent to the IC. This collateral projection was revealed by placing different retrograde tracers into the IC and the MG, which yielded double-labeled cells in the cochlear nucleus. These same injections labeled many cells in other brainstem auditory nuclei. We report here that many of these cells also send branching axonal projections to innervate both the auditory midbrain and the auditory thalamus. While these cells are outnumbered by those in the “lemniscal” auditory pathways, their widespread distribution and possibilities for rapid transmission of information to higher auditory centers suggests that they could play an important role in auditory function.

## Materials and methods

All procedures were conducted in accordance with the Institutional Animal Care and Use Committee of the Northeast Ohio Medical University and NIH guidelines. Results are described from 15 adult pigmented guinea pigs (Elm Hill Labs; Chelmsford, MA, USA) of either gender weighing 258–729 g. Efforts were made to minimize the number of animals and their suffering. Data from some of these animals has been presented previously (e.g., Motts and Schofield, 2010, 2011; Schofield et al., 2014); those publications describe labeling of cells in the cochlear nucleus or the cholinergic cells of the pontomesencephalic tegmentum. The present work presents unpublished data on labeled cells in the superior olivary complex and lateral lemniscal regions.

### Surgery

Details of the surgical procedures have been published previously (e.g., Motts and Schofield, 2010). Briefly, each animal was anesthetized with isoflurane in oxygen. Ointment (Moisture Eyes PM ophthalmic ointment, Bausch and Lomb, Rochester, NY, USA) was applied to each eye to protect the cornea from drying. Atropine sulfate (0.08 mg/kg i.m.) was given to minimize respiratory secretions and Ketofen (ketoprofen, 3 mg/kg i.m.; Henry Schein, Melville, NY 11747, USA) was given for postoperative analgesia. The animal was positioned in a stereotaxic frame. Body temperature was maintained with a feedback-controlled heating pad. Sterile instruments and aseptic techniques were used for all surgical procedures. An incision was made in the scalp and the surrounding skin was injected with Marcaine (0.25% bupivacaine with epinephrine 1:200,000; Hospira, Inc., Lake Forest, IL, USA), a long-lasting local anesthetic. A dental drill was used to make a craniotomy over the desired target coordinates. After the tracer injection, the craniotomy was filled with Gelfoam (Harvard Apparatus, Holliston, MA, USA) and the scalp was sutured. The animal was then moved to a clean cage and monitored until it could walk, eat and drink without difficulty.

### Retrograde tracers

Four fluorescent tracers were used: red fluorescent RetroBeads (“red beads”; RB; Luma-Fluor, Inc., Naples, FL, USA) and green fluorescent RetroBeads (“green beads”; GB; Luma-Fluor); FluoroGold (FG; FluoroChrome, Inc., Englewood, CO, USA) and Fast Blue (EMS-Chemi GmbH, Gross Umstadt, Germany). Large injections were made with a Hamilton microsyringe (1 μl; Hamilton, Reno, NV, USA). Each syringe was dedicated to a single tracer. Large injections into the MG were accomplished by depositing tracer at 1–4 sites within the target (Table 1). The number of deposits and the volume deposited at each site were designed to account for the diffusibility of each tracer, with the goal of filling as much of the target as possible while limiting spread to surrounding areas (Schofield, 2008). In four animals (the first four listed in Table 1), GB tracer was deposited with a 1 μl microsyringe at 4–6 sites within the IC (total volume 0.6–0.75 μl).

**Table 1:**
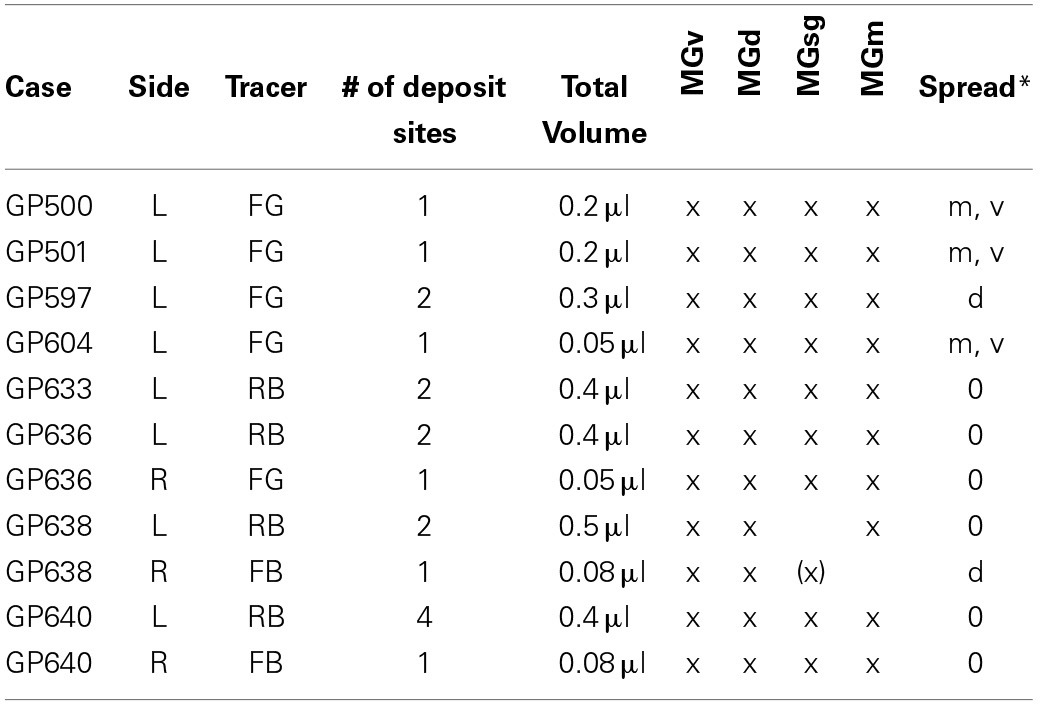
**Large injections into the MG**.

Summary of tracer injection parameters and distribution of deposit sites. Fluorescent tracers were deposited into left (L) or right (R) medial geniculate nuclei (MG). Deposits were made at 1 or more sites (# of deposit sites) in each MG. Total volume = volume of tracer deposited within the MG on the indicated side. For each MG, the subdivisions that were included in the deposit sites are indicated: “x” indicates substantial deposit within the subdivision, “(x)” indicates only minor encroachment of the tracer into that subdivision. An empty space (e.g., under MGm for Case GP638 R), indicates no spread of tracer into that subdivision. ^*^Spread: In some cases, the tracer spread to surrounding nuclei, either medial and ventral to MG (m, v) or dorsal (d) to MG, as indicated. “0” indicates no significant spread into surrounding nuclei. FB, Fast Blue; FG, FluoroGold; GB, green beads; MGd, MGm, MGsg, MGv, dorsal, medial, suprageniculate, and ventral subdivisions of the medial geniculate nucleus; RB, red beads.

Small injections directed at single subdivisions of the MG were accomplished in most cases with a glass micropipette (tip inside diameter 25–35 μm) attached to a Nanoliter Injector (World Precision Instruments, Sarasota, FL, USA) (Table 2). In two animals (GP718 and GP719), FG was injected into the MG with a microsyringe (with the intent of making a large injection), but for unknown reasons the result in each case was a small injection confined to a single MG subdivision (Table 2, Cases GP718, GP719). These cases were analyzed with the others that had small, confined injections.

**Table 2:**
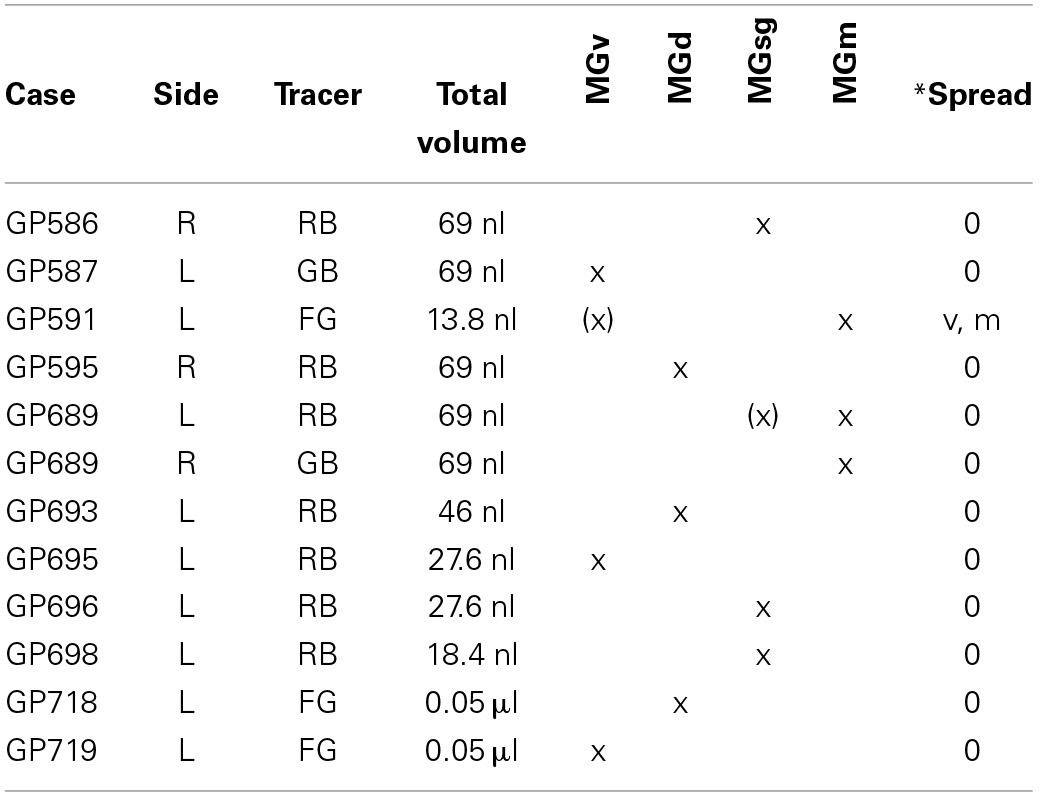
**Small injections into the MG**.

Summary of tracer injection parameters and distribution of deposit sites for cases with small injections directed at individual MG subdivisions. Fluorescent tracers were deposited into left (L) or right (R) medial geniculate nuclei (MG). All tracers were deposited at a single location within the MG. Total volume = volume of tracer deposited within the MG on the indicated side. Volumes indicated in nanoliters (nl) represent injections made with a Nanoliter Injector (see text); volumes indicated in microliters (μl) were made with a 1 μl microsyringe (see text for discussion). For each MG, the subdivisions that were included in the deposit sites are indicated: “x” indicates substantial deposit within the subdivision, “(x)” indicates only minor encroachment of the tracer into that subdivision. An empty space indicates no spread of tracer into that subdivision. ^*^Spread: In Case GP591, the tracer spread medial and ventral to the MGm; none of the other cases had significant tracer spread outside the MG.

In 4 cases, FluoroRuby (tetramethylrhodamine dextran) was injected into the auditory cortex as part of a separate study (to be described in a separate report). FluoroRuby fluoresces in red and was used only in cases with FG and GB injections (in the MG and IC, respectively) so that the tracers could be distinguished without difficulty.

### Perfusion and tissue processing

Five to twenty-five days after surgery, the animal was deeply anesthetized with isoflurane and perfused transcardially with Tyrode's solution followed by 250 ml of 4% paraformaldehyde in 0.1M phosphate buffer, pH 7.4 and then by 250 ml of the same fixative with 10% sucrose. The brain was removed and stored at 4°C in fixative with 25–30% sucrose for cryoprotection. The following day the cerebellum was removed and the remaining tissue block was trimmed with transverse cuts posterior to the cochlear nucleus and anterior to the auditory cortex. The tissue was frozen and cut on a sliding microtome into 40 or 50 μm thick transverse sections that were collected in six sets.

### Data analysis

MG subdivisions were identified by their patterns of staining with cytochrome oxidase (Anderson et al., 2007). IC subdivisions were identified by the differential expression of brain nitric oxide synthase (Coote and Rees, 2008). The nuclei of the superior olivary complex were identified according to Schofield and Cant (1991, 1999). Subdivisions of the ventral nucleus of the lateral lemniscus are based on Schofield and Cant (1997). Remaining areas were identified by comparison with an atlas of the rat brain (Paxinos and Watson, 1998).

The location and extent of each injection site was determined by comparison of the tracer deposit with borders of MG subdivisions identified in sections stained for cytochrome oxidase (Anderson et al., 2007). Labeled cells in the superior olivary complex and in areas in or around the lateral lemniscus were plotted with a Neurolucida reconstruction system (MBF Bioscience, Williston, VT, USA) attached to a Zeiss Axioplan II microscope (Carl Zeiss MicroImaging, Inc., Thornwood, NY, USA). Cells containing one or two different fluorescent tracers were readily distinguished from one another and from unlabeled cells. Cells that contained a particular tracer were interpreted as projecting to the injection site. Cells that contained two tracers were interpreted as cells that send branching axons (i.e., collateral projections) to the two different injection sites. Each combination of tracers was plotted with a unique marker. In general, the labeled cells were studied in every sixth section or, in cases with relatively few labeled cells, in every third section. Five cases (GP500, GP501, GP597, GP609, GP640) with large injections and robust retrograde labeling were chosen for quantitative analysis of the distribution of labeled cells in the subcollicular nuclei. Data from the dorsal and ventral cochlear nuclei in some of these cases (all but GP640) were described previously (Schofield et al., 2014); the present data were supplemented with data from GP640 to allow comparison of the number of labeled cells in the cochlear nuclei with numbers of cells in the nuclei described in the present study. Four of these cases (excepting GP640) also received injections of a second retrograde tracer into the IC ipsilateral to the MG injection; these cases were used for quantitative analysis of the number and distribution of double-labeled cells. Every area that contained MG-projecting cells also contained IC-projecting cells, and the latter population often greatly outnumbered the MG-projecting cells. In order to quantify the collateral projections, the double-labeled cells were calculated as a percentage of all the MG-projecting cells in the same nucleus. Both sets of quantitative analyses are based on cell counts from every sixth section through the relevant nuclei.

### Illustration and photography

Plots showing the distribution of labeled cells were created with Neurolucida software (MBF Bioscience) and refined with Adobe Illustrator (Adobe Systems, Inc., San Jose, CA, USA). Photomicrographs were captured using either a Zeiss AxioImager Z1 fluorescence microscope and AxioCam MRm or HRc cameras (Zeiss) or a Zeiss Axioskop fluorescence microscope and Magnafire camera (Optronics, Goleta, CA, USA). Adobe Photoshop (Adobe Systems) was used to add scale bars, crop images, erase background around tissue sections, adjust intensity levels and colorize monochrome images.

## Results

We present the results in three sections. First, we describe the distribution of subcollicular cells after large injections into the MG. Second we describe the distribution of labeled cells after injections of tracer confined to individual subdivisions of the MG. Third, we describe the results of dual injections of different tracers into the MG and the IC to identify cells that send collateral projections to both targets. We describe the injection sites, the distribution of the double-labeled cells and then the morphology of the MG-projecting cells (single and double-labeled).

We examined cases with a wide range of survival times (5–25 days) to see if additional time for retrograde transport (as compared to the commonly used 3–6 days) affected the results. Our results appeared similar across cases with short (5–7 days) or long (11–25 days) survival times, suggesting that the shorter survival times were adequate for analyzing the pathways under study. Results across tracers were also similar, with the exceptions (as described in previous studies) that red and green beads tend to label more cells than FG or FB (with equivalent injection sites), but that the latter tracers reveal somatic (and sometimes dendritic) morphology to a greater extent than the beads (discussed in Schofield et al., 2007; Schofield, 2008). The results from the various tracers are described together in the following sections.

### Distribution of cells that project to the MG

#### Injection sites

**Figure 1:**
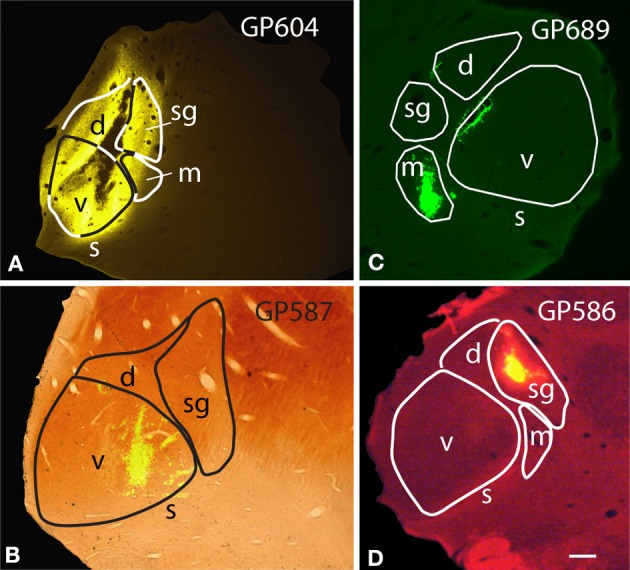
**Photomicrographs of representative injection sites in the medial geniculate nucleus (MG). (A)** Large injection of FluoroGold that involved all MG subdivisions. **(B)** Small injection of green beads confined to the ventral subdivision of the MG and visualized in a section that was stained for cytochrome oxidase activity (combined fluorescence and brightfield image). **(C)** Small injection of green beads confined to the medial subdivision of MG (m). Additional green fluorescence is seen around the margins of a blood vessel along the dorsomedial border of the ventral MG (v); this represents spread of beads that does not result in retrogradely labeled cells. **(D)** Small injection of red beads into the suprageniculate MG (sg). Scale bar in **(D)** = 0.5 mm and applies to **(A–D)**. Transverse sections; dorsal is up; lateral is to the left in **(A,B,D)**, and to the right in **(C)**. Panels **(A,B,D)** were originally published in Schofield et al. (2014).

In order to maximize the chances of finding MG-projecting cells, we made large injections of different tracers into the MG. For most of the experiments, we made multiple deposits of tracer at different locations in the MG. The resulting injection sites routinely included large portions of all MG subdivisions (Figure 1A). In some cases, tracer spread into nearby parts of the anterior pretectal nucleus, the lateral posterior or the posterior thalamic nuclei (all located medial to the MG) or the dorsal lateral geniculate nucleus (dorsal to the MG) (Table 1). We excluded any experiments where the injection spread caudally into the superior colliculus or the nucleus of the brachium of the IC. Smaller injections were typically confined to a single MG subdivision (Table 2). Figure 1 shows representative injection sites in the MGv (Figure 1B), the MGm (Figure 1C) and the MGsg (Figure 1D). The smaller injections usually labeled fewer cells than the large injections, but otherwise were consistent with the results of large injections. This suggests that spread of tracer into regions surrounding the MG did not affect the conclusions. In addition, the results were similar across animals with the same tracer injected and across animals with different tracers.

#### Distribution of MG-projecting cells after large injections in the MG

Labeled cells were present in many areas, including ipsilateral and contralateral IC and non-auditory nuclei such as the spinal trigeminal nucleus and the pontomedullary cholinergic nuclei (pedunculopontine and laterodorsal tegmental nuclei). We will restrict our discussion to subcollicular nuclei traditionally considered part of the auditory pathways. Labeled cells in the dorsal and ventral cochlear nuclei have been described previously (Schofield et al., 2014).

**Figure 2:**
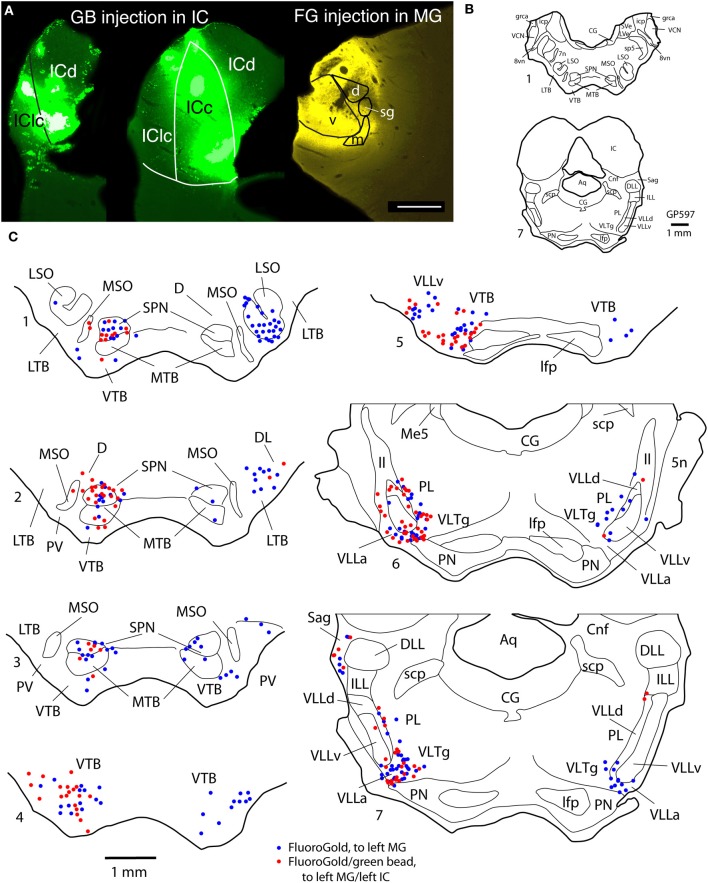
**Photographs and plots showing injection sites and the resultant distribution of labeled cells in transverse sections through brainstem auditory nuclei after injection of FluoroGold (FG) into the left MG and green beads (GB) into the left IC. (A)** Photomicrographs showing dual injections of green beads into the IC and FluoroGold into the MG. Green beads were deposited at multiple sites in the left IC as shown here in a very caudal section (left section) and a more centrally located section (middle section). IC subdivisions are shown, illustrating the spread of green beads into the lateral cortex (IClc), the dorsal cortex (ICd) and the central nucleus (ICc). The right-most section shows a large injection of FluoroGold into the left MG in the same animal. Case GP597. Scale bar = 1 mm. This panel was originally published in Schofield et al. (2014). **(B,C)** Plots showing the distribution of labeled cells in subcollicular nuclei after the injections illustrated in **(A)**. **(B)** Drawings of sections to provide orientation and to indicate nuclear outlines for enlargements shown in **(C)**. Numbers at the lower left of each section correspond to section numbers in **(C)**. **(C)** Enlargements of the superior olivary complex and lateral lemniscal regions showing locations of labeled cells. Each symbol represents one or more labeled cells. Sections are numbered from caudal to rostral and are spaced 300 μm apart. See list for abbreviations.

**Figure 3:**
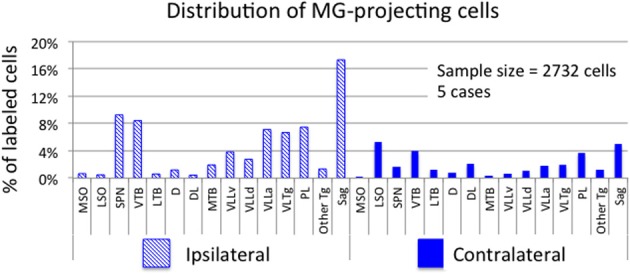
**Graph showing the distribution of labeled cells in subcollicular auditory nuclei after injection of a retrograde tracer into the medial geniculate nucleus (MG).** Each bar shows the percentage of cells (averaged across 5 cases) located in each nucleus. Cross-hatched bars show values for nuclei ipsilateral to the MG injection site and solid bars show values for contralateral nuclei. “Other Tg” indicates scattered cells in intermediate and dorsal nuclei of the lateral lemniscus as well as rostrally and caudally adjacent areas not included in the paralemniscal area. See list for other abbreviations.

Figure 2 shows plots of labeled cells in a representative case following a large injection of FG into the left MG. This case also included a large injection of GB into the left IC to label collicular-projecting cells. A subset of the labeled cells contained both FG and GB, indicating collateral projections to the MG and the IC. We first describe the distribution of the MG-projecting cells (with or without collaterals to the IC). FG-labeled cells (red and blue symbols in Figure 2) were present bilaterally in a majority of the brainstem auditory nuclei. Figure 3 shows a quantitative summary of the distribution of labeled cells in these nuclei, averaged across 5 experiments chosen for robust retrograde labeling. Overall, 66% of the labeled cells were found ipsilateral to the MG injection.

Most of the nuclei in the superior olivary complex contained labeled cells. On the ipsilateral side, labeled cells were most numerous in the superior paraolivary nucleus (SPN) and the ventral nucleus of the trapezoid body (VTB), with additional cells in the medial nucleus of the trapezoid body (MTB) and other periolivary nuclei. The only olivary nucleus that never contained labeled cells was the posteroventral periolivary nucleus, a small nucleus located between the ventral and lateral nuclei of the trapezoid body (Schofield and Cant, 1991). Labeled cells were also present in the two principal nuclei of the superior olive, the medial and lateral superior olivary nuclei (MSO and LSO), but these were outnumbered by labeled periolivary cells. On the contralateral side, labeled cells were fewer but still spread across most olivary nuclei (Figures 2, 3). The notable exceptions were a lack of labeled cells in the MSO and a relatively larger percentage in the LSO (Figures 2, 3).

FG-labeled cells were also present in and around the nuclei of the lateral lemniscus. The cells were present bilaterally in similar distributions, with a greater number of cells on the ipsilateral side. One group of cells was concentrated in and around the ventral nucleus of the lateral lemniscus (VLL). Some of these cells were located clearly within the VLL ventral subdivision (VLLv; see Schofield and Cant, 1997 for definition of the VLL subdivisions) but similar cells were distributed rather smoothly across the border between the VLL and the VTB and into the rostro-ventrally adjacent anterior division of VLL (VLLa) (Figure 2, section 4 to section 7). Additional FG-labeled cells were located in the VLL dorsal subdivision (VLLd). A second concentration of labeled cells was found in the reticular formation medial to the ventral end of the VLL, in a region called the ventrolateral tegmental nucleus (VLTg). Labeled cells were scattered more dorsally, in the so-called paralemniscal area. A third concentration of FG-labeled cells associated with the lateral lemniscus was found in the sagulum, located between the dorsal nucleus of the lateral lemniscus and the lateral surface of the brainstem (Figure 2, section 7). Although FG-labeled cells were absent from the contralateral sagulum in the case illustrated in Figure 2, they were present in this area in other cases (Figure 3). FG-labeled cells were occasionally observed in the dorsal or intermediate nuclei of the lateral lemniscus on either side of the brain (indicated as “other tegmental areas,” “Other Tg” in Figure 3).

### Distribution of cells that project to specific MG subdivisions

We examined results from 12 cases in which the tracer deposit was restricted to one (or, in 2 cases, two) MG subdivisions (Table 2). An injection confined to the medial MGm produced the highest number of labeled cells and came closest to matching the labeling after large injections of the MG. The distribution of labeled cells was similar in a second case where the deposit site was centered on the MGm but encroached on the suprageniculate subdivision (MGsg). Injections into other subdivisions labeled fewer cells; importantly, well-labeled cells were observed in several areas in these cases (e.g., cochlear nucleus, pedunculopontine tegmental nucleus), suggesting that the small number of labeled cells in olivary and lemniscal areas was not due to failure of tracer transport. Tracer deposits confined to the MGsg labeled very few cells; most of these were located in the ipsilateral paralemniscal area. Injections confined to the ventral (MGv) subdivision also labeled very few cells. These cells were scattered among the olivary and lemniscal regions described above, but were too few to establish a pattern. Finally, injections confined to the dorsal subdivision (MGd) labeled slightly more cells, but fewer than injections into the MGm. These cells were scattered among the same nuclei described above; the specific pattern varied across the 3 cases, with one case having labeled cells only in the SOC, a second case with cells mostly in the sagulum and the third case with cells spread evenly across all regions. The differences were not readily attributable to differences in the injection site locations. Taken together, the results of the small injections suggest that the MGm is the main target of the subcollicular projections, with the MGd a secondary target. The MGv and MGsg appear to receive projections from relatively few subcollicular auditory cells.

### Distribution of cells that project to the MG and the IC

#### Injection sites

Figure 2A shows representative injection sites in the MG and the inferior colliculus (IC) from one of the animals that received dual injections of FG and GB to identify cells with collateral projections to the injected targets. The injection illustrated in Figure 2 was one of the largest, and spread dorsal to the MG. The IC injections routinely involved the central nucleus, the lateral cortex and the dorsal cortex and, in some cases, spread rostrally into the intercollicular tegmentum (also called rostral cortex). None of the injections spread outside the borders of the IC or across the midline into the contralateral IC. While no individual injection site encompassed an entire IC, the labeling across the cases suggests that all major parts of the IC, and thus the ascending pathways, were included in the analysis. The IC injections labeled cells in auditory and non-auditory areas (e.g., cochlear nuclei, trigeminal nuclei, pedunculopontine and laterodorsal tegmental nuclei), including the superior olivary and lemniscal regions that are the focus of the present report.

#### Distribution of double-labeled cells that project to both MG and IC

Figure 2C shows the distribution of cells labeled by the FG injection into the left MG (shown in Figure 2A). The double-labeled cells (red circles) are interpreted as cells that send branching axonal projections to the IC and the MG. The great majority of such cells were located ipsilateral to the IC and MG injections. These cells were concentrated in the SPN, VTB, and VLL (including VLLa, VLLv, and VLLd) and the VLTg. In the VTB, the double-labeled cells were spread across much of the nucleus, but were most numerous rostrally. Finally, double-labeled cells were numerous in the sagulum (Figure 2, section 7). Additional double-labeled cells were observed in the MSO, LSO, and MTB. Many cells, not illustrated, were labeled by green beads. In most regions described, these cells substantially outnumbered cells that contained FG (with or without green beads).

Fewer double-labeled cells were present contralateral to the injections. These cells were scattered in the lateral periolivary regions (in the lateral nucleus of the trapezoid body and in the dorsolateral periolivary nucleus) and in areas surrounding the VLLv (namely, in the VLLa, VLLd and the paralemniscal area).

### Morphology of MG-projecting cells

#### Cells in the superior olivary complex

The olivary nuclei contain a variety of cell types that can be distinguished by soma morphology (Schofield and Cant, 1991). In some cases the morphological differences correlate with projections of the cells (Schofield, 1991, 1994; Schofield and Cant, 1992). We provide a brief description of the morphology of cells labeled by the MG injections or by the dual injections into the MG and the IC.

**Figure 4:**
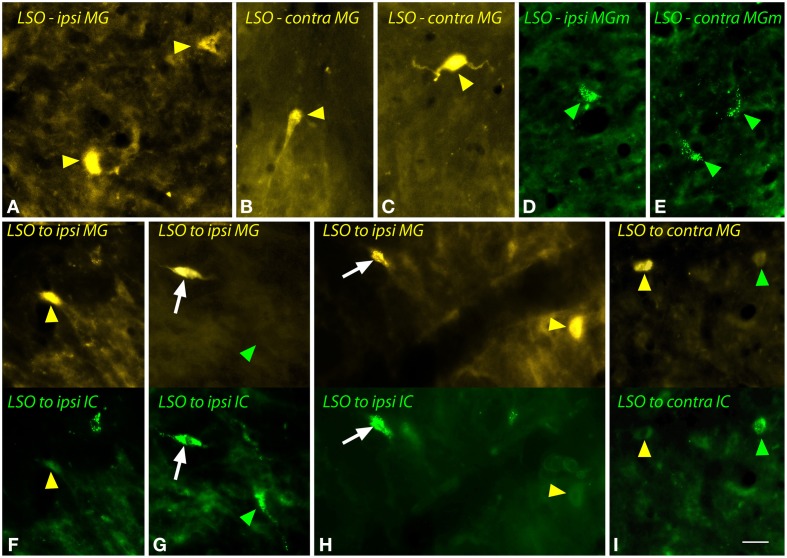
**Photomicrographs showing labeled cells in the lateral superior olivary nucleus (LSO) after injections into the medial geniculate nucleus (MG) and the inferior colliculus (IC). (A–C)** Cells labeled by a large injection of FluoroGold into the left MG (GP597). Labeled cells (yellow arrowheads) are shown ipsilateral **(A)** or contralateral **(B,C)** to the injection site. **(D,E)** Cells labeled by a small injection of green beads into the right MG medial subdivision (MGm). Labeled cells (green arrowheads) are shown ipsilateral **(D)** and contralateral **(E)** to the injection site (GP689). **(F–I)** Single- and double-labeled cells in the LSO after injections of FluoroGold into the left MG and green beads into the left IC (GP597; injection sites shown in Figure 2A). For each panel, the upper half shows cells labeled with FluoroGold and the lower half shows the same area imaged for green beads. White arrows indicate cells that are labeled with both tracers, indicating collateral projections to the two injection sites. Other cells are labeled with only FluoroGold (yellow arrowheads) or green beads (green arrowheads). **(F–H)** show cells in the LSO ipsilateral to the injections; **(I)** shows cells contralateral to the injections. Scale bar = 20 μm and applies to all panels. Transverse sections; dorsal is up; lateral is to the left (**A,E,F–H**) or to the right (**B–D,I**). Abbreviations: contra, contralateral; ipsi, ipsilateral.

The body of the LSO contains fusiform (bipolar) cells and multipolar cells. Figure 4 shows examples of the cells labeled by a large FG injection into the MG (Figures 4A–C) or a small injection into the MGm (Figures 4D,E). Labeled fusiform cells were observed more often than multipolar cells, but both types were labeled on both sides of the brain. Following dual injections into left MG and left IC, double-labeled LSO cells were readily identified (Figures 4G,H). They were usually fusiform. FG-labeled cells were found near GB-labeled cells on both sides (Figures 4F–I).

**Figure 5:**
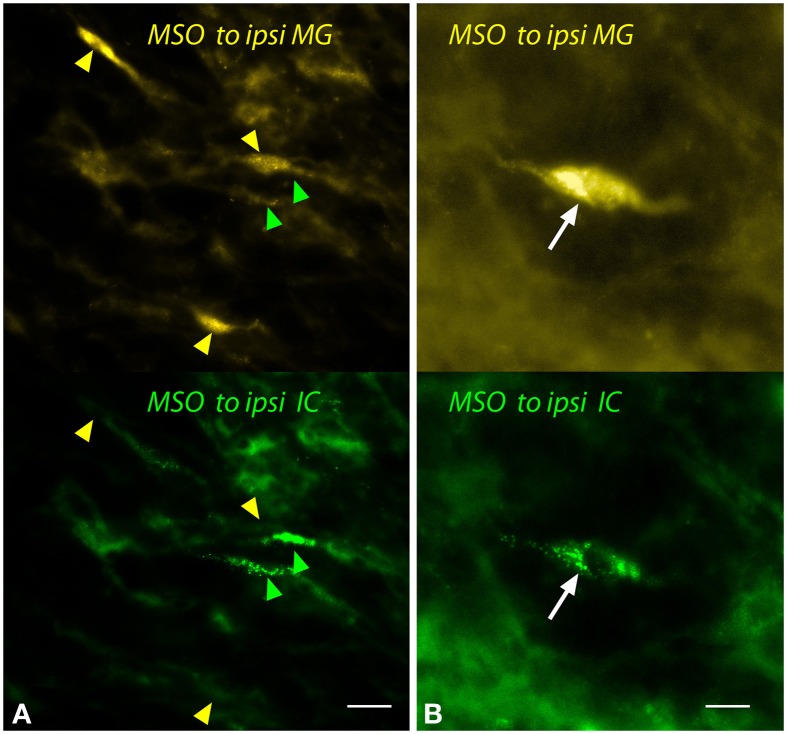
**Photomicrographs showing labeled cells in the medial superior olivary nucleus (MSO) after injections into the medial geniculate nucleus (MG) and the inferior colliculus (IC).** Panels show FluoroGold (upper image) and green beads (lower image). **(A)** Single-labeled cells that contained only FluoroGold (yellow arrowheads) or only green beads (green arrowheads). **(B)** A double-labeled cell (white arrow) contained both tracers. Transverse sections; dorsal is up; lateral is to the left. Scale bar = 20 μm **(A)** or 10 μm **(B)**. ipsi, ipsilateral.

Despite the relatively small number of MSO cells labeled by any MG injection, both single- and double-labeled cells were observed frequently (Figure 5). These cells showed the fusiform morphology typical of MSO cells. Single-labeled cells, projecting to the MG or to the IC, were found near one another (Figure 5A).

**Figure 6:**
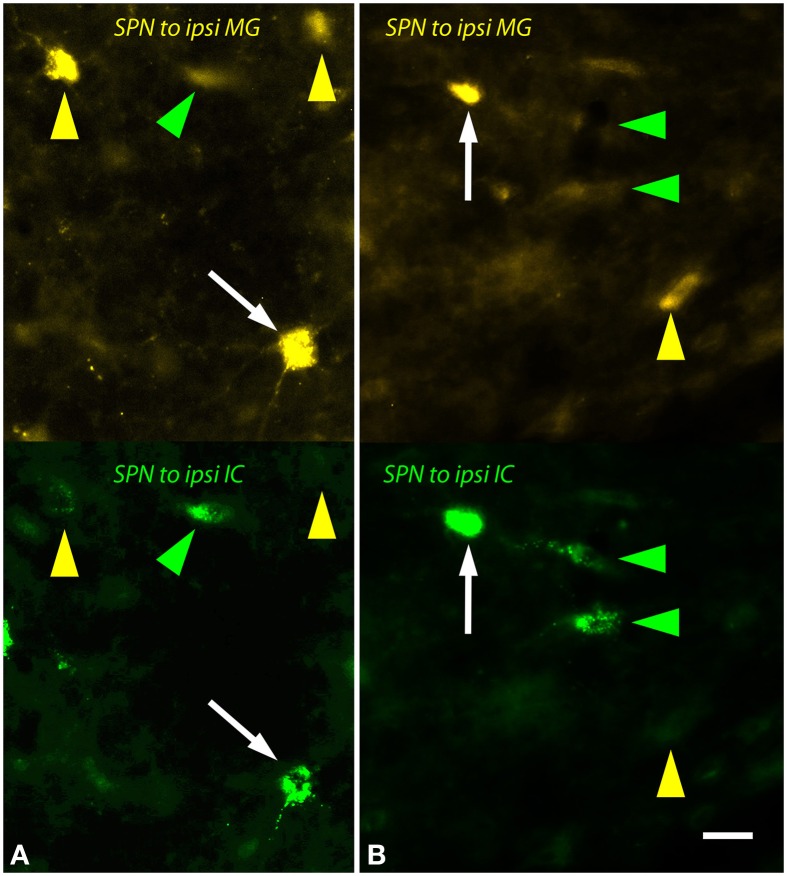
**Photomicrographs showing labeled cells in the left superior paraolivary nucleus (SPN) after injections into the medial geniculate nucleus (MG) and the inferior colliculus (IC).** Each panel shows FluoroGold (upper half) and green beads (lower half). **(A,B)** Double-labeled cells are numerous (white arrows). Additional cells contained only FluoroGold (yellow arrows) or only green beads (green arrowheads). Transverse sections; dorsal is up; lateral is to the left. Scale bar = 20 μm. ipsi, ipsilateral.

The ipsilateral SPN contained many single and double-labeled cells (Figure 6). Many of these cells were multipolar with relatively round somas (e.g., Figure 6A) while others had more elongated somas (e.g., Figure 6B). The contralateral SPN contained fewer MG-projecting cells. These were almost exclusively single-labeled. Morphologically they appeared similar to the MG-projecting cells in the ipsilateral SPN.

**Figure 7:**
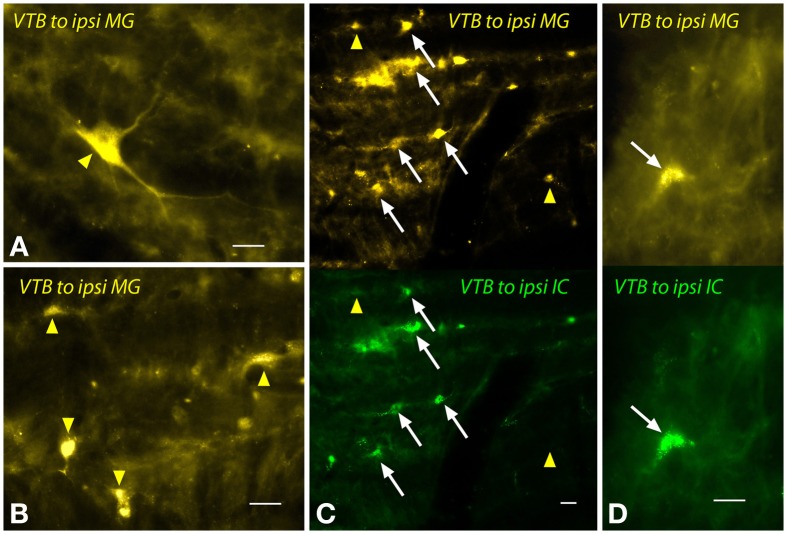
**Photomicrographs showing labeled cells in the ventral nucleus of the trapezoid body (VTB) after injections into the medial geniculate nucleus (MG) and the inferior colliculus (IC). (A,B)** Labeled cells (yellow arrowheads) after a large injection of FluoroGold into the ipsilateral MG (GP597). **(C,D)** Single- and double-labeled cells in the left VTB after injections of FluoroGold into the left MG and green beads into the left IC (GP597). For each panel, the upper half shows cells labeled with FluoroGold and the lower half shows the same area visualized to see green beads. White arrows indicate cells labeled with both tracers. Other cells are labeled with only FluoroGold (yellow arrowheads) or green beads (unmarked). Scale bar = 20 μm. All panels: transverse sections; dorsal is up; lateral is to the left. Abbreviations: contra, contralateral; ipsi, ipsilateral.

The labeled cells in the VTB were morphologically heterogeneous (Figure 7). Large multipolar cells (~15–25 μm diameter) were labeled frequently by MG injections (Figure 7A), but not by IC injections. These cells were observed only ipsilateral to the MG injection and were concentrated in the rostral part of the VTB. A larger number of labeled cells had medium to small somas with fusiform or multipolar shapes (e.g., Figure 7B). Similar cells were labeled on the contralateral side. On the ipsilateral side, many of these cells were double-labeled by the dual injections (Figures 7C,D). Other cells were single-labeled with FG (Figure 7, yellow arrowheads) or with GB (not shown).

**Figure 8:**
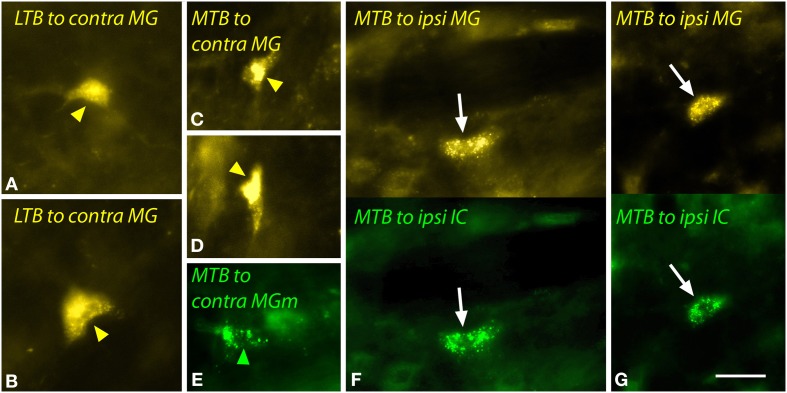
**Photomicrographs showing labeled cells in the lateral nucleus of the trapezoid body (LTB) or the medial nucleus of the trapezoid body (MTB) after injections into the medial geniculate nucleus (MG) and the inferior colliculus (IC). (A,B)** Cells (yellow arrowheads) in the right LTB labeled by a large injection of FluoroGold into the left MG (GP597). **(C,D)** Labeled cells (yellow arrowheads) in the right MTB after a large injection of FluoroGold into the left MG (GP597). **(E)** Cell (green arrowhead) in the left MTB labeled by a small injection of green beads into the right MG medial subdivision (MGm) (GP689). **(F,G)** Single- and double-labeled cells in the left MTB after injections of FluoroGold into the left MG and green beads into the left IC (GP597). For each panel, the upper half shows cells labeled with FluoroGold and each lower half shows the same area visualized to see green beads. White arrows indicate cells that are labeled with both tracers. Scale bar = 20 μm and applies to all panels. Transverse sections; dorsal is up; lateral is to the right **(A–D)** or left **(E–G)**. Abbreviations: contra, contralateral; ipsi, ipsilateral.

The LTB and MTB also contained labeled cells. The LTB cells, located predominantly contralaterally, included relatively large multipolar cells (Figures 8A,B) as well as smaller cells; some of the latter were double-labeled. In the MTB, the majority of MG-projecting cells were located ipsilaterally, but the cells were morphologically similar on both sides. These cells had small elongated somas (Figures 8C–G). Injections restricted to the MGm labeled similar cells in the MTB (Figure 8E). Double-labeled cells after dual tracer injections into the MG and the IC were observed frequently (Figures 8F,G).

#### Cells in and around the nuclei of the lateral lemniscus

**Figure 9:**
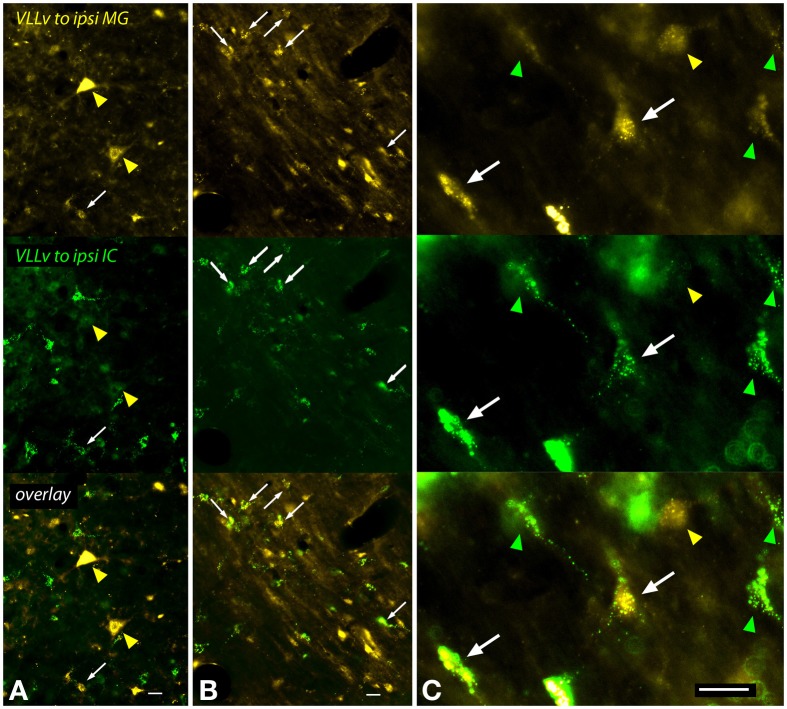
**Photomicrographs showing labeled cells in the left ventral subdivision of the ventral nucleus of the lateral lemniscus (VLLv) after injections into the left medial geniculate nucleus (MG) and the left inferior colliculus (IC) (GP597).** Each panel is divided into thirds, with the top part showing FluoroGold label, the middle part showing green bead label and the bottom part showing an overlay of the two images. **(A,B)** Low magnification images to show both the large number of labeled cells and the range of sizes of the labeled cell bodies. Double-labeled cells were present (white arrowheads), but did not include the largest cells, which were labeled only by FluoroGold (yellow arrowheads in **A**). **(C)** Higher magnification images to show double-labeled cells (white arrows) interspersed among cells labeled with only FluoroGold (yellow arrowhead) or green beads (green arrowheads). The double-labeled cells are multipolar cells, one with a multipolar soma (right arrow) and one with an elongate soma (left arrow). The FG-only cell (yellow arrowhead) has a round soma and may be a globular or multipolar cell. Scale bar = 20 μm. All panels: transverse sections; dorsal is up; lateral is to the left. Abbreviations: ipsi, ipsilateral.

A large number of cells in and around the VLL were labeled after an injection into the MG (Figure 9). The VLLv contains several cell types, including giant cells as well as smaller cells that are globular (with round cell bodies) or multipolar (Schofield and Cant, 1997). Multipolar cells with elongate or more rounded multipolar somas were labeled frequently, and many of these cells were double-labeled after dual tracer injections (Figure 9C, white arrows). Giant cells, which are relatively rare in the VLLv, were labeled by MG injections. The giant cells were not labeled by IC injections, and were never double-labeled in cases with dual injections (Figure 9A, yellow arrowheads).

**Figure 10:**
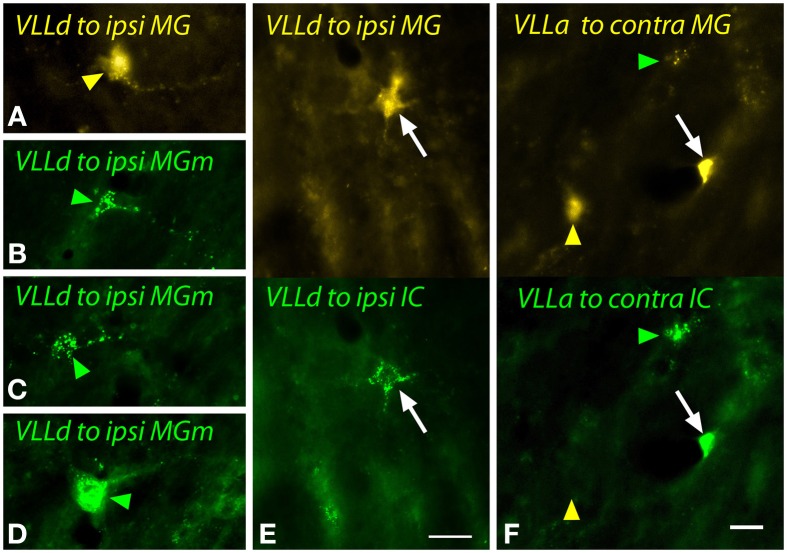
**Photomicrographs showing labeled cells in the dorsal (VLLd) and anterior (VLLa) subdivisions of the ventral nucleus of the lateral lemniscus after injections into the medial geniculate nucleus (MG) and the inferior colliculus (IC). (A)** Cell in the left VLLd labeled after a large injection of FluoroGold into the left MG (GP597). **(B–D)** Cells in the right VLLd after injection of green beads into the right MG medial subdivision (MGm; GP689). **(E)** Double-labeled cell (white arrow) in the left VLLd after injections of FluoroGold into the left MG and green beads into the left IC (GP597). **(F)** Single- and double-labeled cells in the right VLLa after injections of FluoroGold into the left MG and green beads into the left IC (GP597). The upper half shows the cells labeled with FluoroGold and the lower half shows the same area imaged to show green beads. Labeling patterns included double-labeled cells (white arrow) as well as cells that contained only FluoroGold (yellow arrowheads) or only green beads (green arrowheads). Scale bar = 20 μm and applies to all panels. Transverse sections; dorsal is up; lateral is to the left **(A,E,F)** or right **(B–D)**. Abbreviations: contra, contralateral; ipsi, ipsilateral.

The VLLd contains giant cells and smaller multipolar cells. Figure 10 shows multipolar cells labeled after a large FG injection into the MG (Figure 10A) or a small GB injection in MGm (Figures 10B–D). Dual injections into MG and IC led to double-labeled multipolar cells mostly ipsilateral to the injections (Figure 10E). Giant cells were not labeled in significant numbers in any of the experiments. Small multipolar cells were also labeled in the VLLa. Following dual injections, the VLLa cells could be single labeled (Figure 10F, arrowheads) or double-labeled (Figure 10F, white arrow).

**Figure 11:**
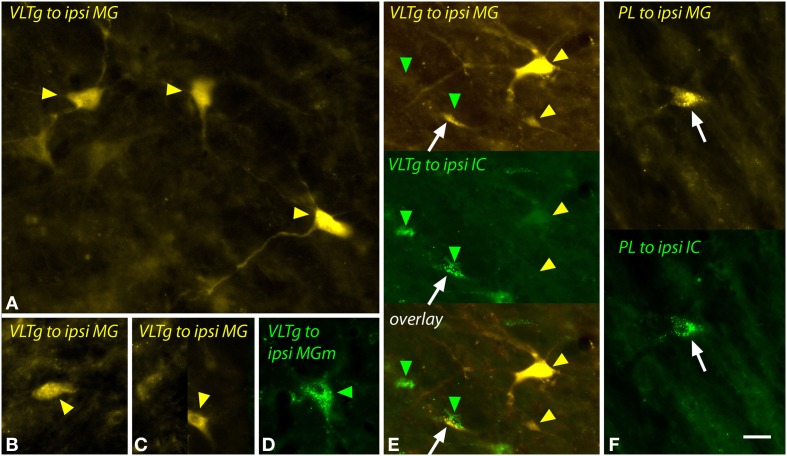
**Photomicrographs showing labeled cells in the ventrolateral tegmental nucleus (VLTg) and the paralemniscal area (PL) after injections into the medial geniculate nucleus (MG) and the inferior colliculus (IC). (A–C)** Cells in the left VLTg labeled after a large injection of FluoroGold into the left MG (GP597). **(D)** Large multipolar cell in the right VLTg labeled by an injection of green beads into the ipsilateral MGm (GP689). (**E)** Single-labeled cells (green and yellow arrowheads) and a double-labeled cell (white arrow) in the left VLTg after injections of FluoroGold into the left MG and green beads into the left IC (GP597). The top panel shows FluoroGold label, the middle panel shows green bead label and the bottom panel shows an overlay of the two images. Most cells were labeled with only FluoroGold (yellow arrowheads) or only green beads (green arrowheads). **(F)** Double-labeled cell (white arrow) in the left paralemniscal area after injections of FluoroGold into the left MG and green beads into the left IC (GP597). The upper half shows the cell labeled with FluoroGold and the lower half shows the same cell imaged to show green beads. Scale bar = 20 μm and applies to all panels. Transverse sections; dorsal is up; lateral is to the left except for panel **(D)**, where lateral is right. Abbreviations: contra, contralateral; ipsi, ipsilateral.

The VLTg was notable because of the large multipolar cells (>20 μm diameter) that were labeled after large injections of FG (Figure 11A) or after a small injection of green beads into the MGm (Figure 11D). Small (~10–20 μm diameter) multipolar and fusiform cells were also labeled in the VLTg (Figures 11B,C). Both large and small cells were labeled bilaterally. Following dual injections into the MG and the IC, the large multipolar cells were labeled only by the MG injections (Figure 11E, upper gold arrowheads) whereas the smaller cells could be double labeled (Figure 11E, white arrow) or could contain either tracer alone (Figure 11E, green arrowheads, lower gold arrowhead).

The paralemniscal area, located along the medial border of the lateral lemniscus, contained a small number of labeled cells after injection into the MG or MGm. These cells were similar to the small multipolar and fusiform cells in the VLTg and, despite their low numbers, could be double-labeled following dual injections in the MG and the IC (Figure 11F, white arrow).

**Figure 12:**
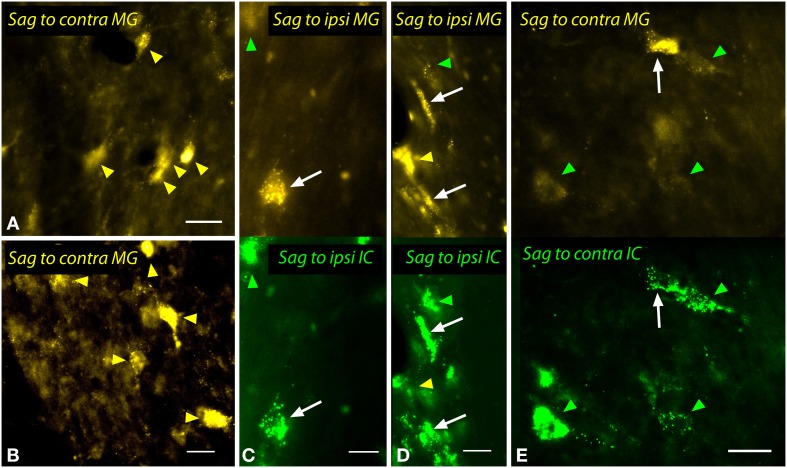
**Photomicrographs showing labeled cells in the sagulum (Sag) after injections into the medial geniculate nucleus (MG) and the inferior colliculus (IC). (A,B)** Cells in the left sagulum labeled after a large injection of FluoroGold into the left MG (GP597). **(C,D)** Double-labeled cells (white arrows) in the left sagulum after injections of FluoroGold into the left MG and green beads into the left IC (GP597). The upper half shows the cells labeled with FluoroGold and the lower half shows the same area visualized to reveal green beads. Single-labeled cells included those with only FluoroGold (yellow arrowheads) or only green beads (green arrowheads). **(E)** Double labeled cell (white arrow) and cells labeled only with GB (green arrowheads) in the right sagulum after dual injections into the left IC and MG. Scale bars = 20 μm. Transverse sections; dorsal is up; lateral is to the right **(A,B,E)** or left **(C,D)**. ipsi, ipsilateral.

The sagulum contained labeled multipolar cells and cells with elongated/fusiform somas bilaterally after injections into the MG (Figure 12). Dual injections into the MG and the IC revealed double-labeled cells bilaterally, including multipolar cells (white arrows in Figures 12C,E) and fusiform cells (white arrows, Figure 12D).

## Discussion

**Figure 13:**
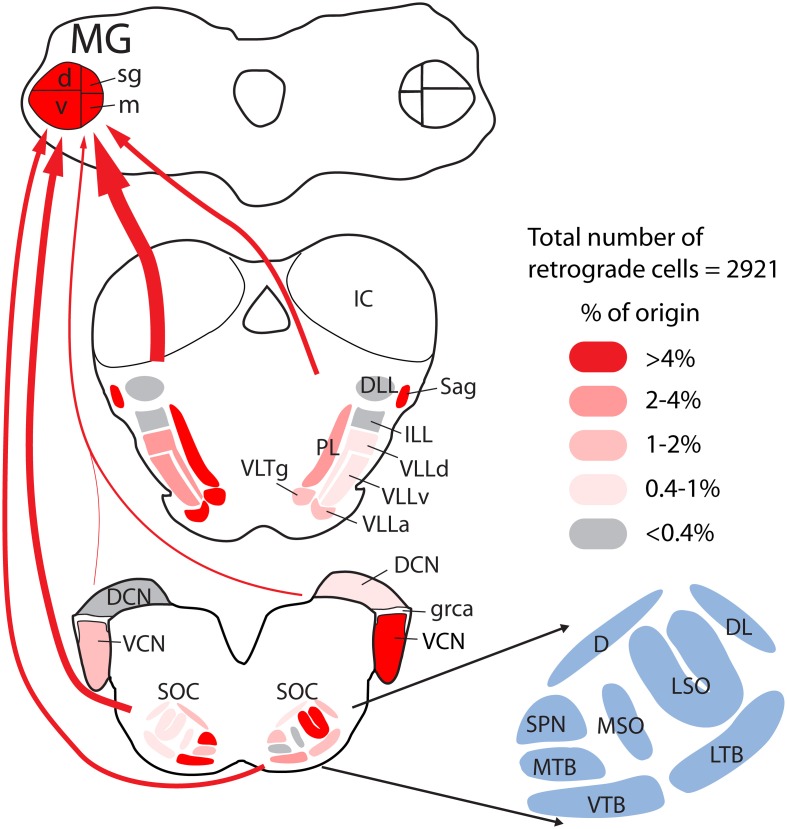
**Quantitative graphical summary of the subcollicular projections to the MG.** The shading of the nuclei and the thickness of the arrows indicate the relative proportion of retrogradely labeled cells in each nucleus/region after large injections of retrograde tracer into the left MG. Data were analyzed as described for Figure 3; additional data for the cochlear nuclei were obtained from a previous study (Schofield et al., 2014) based on the same cases used presently. The percentages of labeled cells in different nuclei ranged from 0 to 16.4%; these values were arbitrarily divided into five categories and shaded as indicated in the legend. Arrows are drawn from groups of nuclei (left or right cochlear nuclei; superior olivary complex, or lemniscal and adjacent tegmental regions), with the thickness of the arrow representing the relative number of labeled cells in each group. Individual nuclei are named in the cross sections or, for the superior olivary complex (SOC), in the adjacent enlargement. See list of abbreviations.

The results presented here demonstrate projections to the MG from a wide array of subcollicular auditory regions. These projections originate from the SOC, NLL, and adjacent tegmental regions and are more extensive than described in any previous study. Figure 13 provides a quantitative summary of the present results combined with the results of our previous study of CN projections to the MG. Together these projections could supply the extralemniscal MG with relatively direct information (i.e., information that did not get processed by the inferior colliculus) about acoustic stimuli. This information is presumably that which underlies the auditory function that remains after bilateral lesions of the brachium of the IC (see Introduction) and perhaps additional functions. We also show that many of the MG-projecting cells send an axon collateral to the IC. Thus, much of the information being transmitted in this pathway to the thalamus does not avoid the IC but gets delivered there as well. In the following sections, we discuss technical considerations and then consider implications for auditory function and for evolution of neural pathways.

### Technical considerations

#### Identification of inputs with retrograde tracing

For identification of overall projections, and for detection of collateral projections, our objective was to maximize the number of labeled cells. We made large injections that sometimes extended beyond the borders of the MG, so we cannot say with certainty that all of the labeled cells project to the MG. However, anterograde tracing studies in guinea pigs and other species show that the MG is the primary or sole thalamic target of direct projections from the cochlear nucleus (Malmierca et al., 2002) and from several regions in or near the lemniscal nuclei (sagulum: Beneyto et al., 1998; paralemniscal regions: Henkel, 1983; Casseday et al., 1989). The results from our small injections, confined to individual MG subdivisions, labeled fewer cells than the large injections but, across cases, produced labeled cells in the same areas as the large injections. This suggests strongly that the MG is a target of projections from each of the nuclei that contained labeled cells. Of course, it is possible that some of the labeled cells have projections to regions outside the MG (including adjacent regions that have auditory or multisensory roles, such as the posterior thalamic nucleus or posterior intralaminar nucleus; see LeDoux et al., 1985). Whether we identified all sources of projections is also a concern, especially with the smaller injections. However, the agreement of results between the small and the large injections, and the agreement across four different retrograde tracers (FluoroGold, Fast Blue, green beads and red beads) suggests that we are unlikely to have missed any substantial source of inputs.

#### Identification of collateral projections

Successful identification of collateral projections requires large injections that, ideally, fill the entire target area. Our large injections into the MG approach this ideal, but our injections into the IC never fully filled the target. In part, this reflects necessary caution to avoid spread of the tracer into adjacent areas such as the superior colliculus, central gray or subcollicular tegmentum that would confound the interpretation of the results. Despite this limitation, we observed many double-labeled cells in every dual injection case. The fact that the IC was not completely labeled, and therefore that many IC-projecting cells were unlabeled, suggests that our analysis of the double-labeled cells is an under-representation of their prominence. Such a conclusion is a common limitation of the method of multiple labeling with retrograde tracers to identify collateral projections (reviewed by Schofield et al., 2007). For this reason, the maximum percentage of double-labeled cells may be more indicative than the average value for assessing the prominence of collateral projections in a pathway (Table 3).

**Table 3:** **Quantitative summary of double-labeled cells**.

	**Ipsilateral**				**Contralateral**			
	**Total #**	**Double-label %**			**Total #**	**Double-label %**		
**Region**	**Cells**	**Max**	**Mean**	**St dev**	**Cells**	**Max**	**Mean**	**St dev**
MSO	15	50%	33%	24%	2	0%	0%	0%
LSO	4	0	0	0	99	50	23	23
SPN	213	55	38	17	23	17	4	8
VTB	172	52	29	17	53	20	11	10
LTB	12	67	34	30	27	63	23	30
D	30	67	36	21	23	0	0	0
DL	7	25	6	13	50	12	6	7
MTB	38	36	26	7	7	0	0	0
VLLv	80	70	45	18	6	25	6	13
VLLd	55	67	36	21	20	17	4	8
VLLa	158	46	32	11	29	18	5	9
VLTg	147	42	24	14	28	17	7	9
PL	149	50	29	16	64	24	11	10
Other Tg	40	100	47	43	35	50	16	24
Sag	426	50	41	8	87	22	8	11

*Cells labeled by the injection of retrograde tracer into the MG were counted in every sixth section through the brainstem in 4 cases (GP500, GP501, GP597, GP609). Cells that were double labeled by both the thalamic injection and the collicular injection were tallied separately. Total number of labeled cells analyzed = 2099, of which 588 were double-labeled. The table shows the number of labeled cells in each nucleus, the maximum percentage of double-labeled cells in that nucleus (Max) across cases, and the mean and standard deviation (St dev) of the percentage of double-labeled cells in each nucleus. See list for abbreviations; “Other Tg”—other tegmental areas, which includes isolated cells in tegmental regions near the lateral lemniscal cells as well as a few scattered cells in the dorsal and intermediate nuclei of the lateral lemniscus*.

### Distribution and morphology of MG-projecting cells

Three earlier studies based on retrograde transport of horseradish peroxidase suggest similarities as well as differences between the present data and results from cats (Henkel, 1983), mustache bat (Casseday et al., 1989) and ferrets (Angelucci et al., 1998). The mustache bat is the most unique, with nearly all subcollicular MG-projecting cells located in a single nucleus, the nucleus of the central acoustic tract (Casseday et al., 1989). The results in cats showed a concentration of cells in the posteromedial subdivision of the VLL (Henkel, 1983), a nucleus in a location similar to the one described in mustache bats. Both cats and ferrets had additional cells in the VLL and the sagulum, and ferrets had labeled cells in the dorsal nucleus of the lateral lemniscus. Anterograde tracing experiments suggest that both ventral and dorsal nuclei of the lateral lemniscus project to the MG in cats (Kudo, 1981). In the superior olivary complex, both ferrets and cats had a few labeled cells in periolivary nuclei (particularly the MTB); these few cells were the only ones labeled in the superior olive of cats, whereas both the MSO and the LSO contained labeled cells in the ferrets. In the following paragraphs, we discuss aspects of cell morphology in selected nuclei where these data help to inform the comparisons between species and, in some instances, have functional implications as well.

The VLTg is a major source of projections to the MG. This nucleus is medial to the VLL, rostral to the VTB and ventrolateral to the oral pontine reticular nucleus (with which a border can be difficult to define). In mustache bats there is a very distinct nucleus in this area comprising large multipolar cells that project to the ipsilateral MG (Casseday et al., 1989). Casseday and colleagues called this the nucleus of the central acoustic tract, recalling earlier descriptions of such a pathway (Papez, 1929a,b). In cats, the same area contains large multipolar cells that project to the MG (ipsilaterally or contralaterally); this area was termed the posteromedial subdivision of the VLL (Henkel, 1983). The present data show projections from large multipolar cells to the ipsilateral or contralateral MG in guinea pigs. These cells are numerous in the VLTg and adjacent parts of the VLL and VTB. In all three species the projection to the thalamus terminates in the extralemniscal MG (the subdivisions outside the MGv). We observed smaller labeled cells among the large cells, suggesting only partial equivalence across species. Following dual injections of different tracers in the IC and the MG, we found that small cells in the VLTg and adjacent areas could be double labeled but the large multipolar cells were labeled only from the MG injection. While there have not been direct tests in other species, evidence from mustache bats indicate that the large cells are not labeled by injections of tracer into the IC, and so couldn't be double-labeled by dual injections (Zook and Casseday, 1982). An “early warning” function was proposed for these large cells based on their projections to the MG and to the superior colliculus (see discussion in Casseday et al., 1989). Whether the large cells serve this or some other function, the similarities across species in location, morphology and projections support the idea of a common function.

The LSO contains populations of cell with very different connections. A large percentage of LSO cells project to the IC and appear to be involved in binaural comparisons and spatial hearing. Other LSO cells project to the cochlea as part of the lateral olivocochlear system (Robertson et al., 1987). We did not check for collateral projections to the cochlea, but we did find LSO cells that project to both the MG and the IC. Under the assumption that the IC-projecting cells contribute to spatial hearing, a similar function may be associated with the LSO projections to the MG.

The MSO, like the LSO, has also been tied closely to binaural comparisons and sound localization. The MSO had significantly fewer MG-projecting cells than did the LSO, but some of these cells were double-labeled in the dual injection experiments. Again, this raises the possibility of spatial hearing as a functional aspect of the subcollicular projections to the MG.

The SPN contained a large collection of MG-projecting cells. The SPN is a prominent source of projections to the IC (Schofield, 1991; Saldaña et al., 2009). Most SPN cells appear to be inhibitory (Helfert et al., 1989; Kulesza and Berrebi, 2000). Both GABAergic and glycinergic cells appear to project to the IC (Saint Marie et al., 1989; Saint Marie and Baker, 1990; González-Hernández et al., 1996). There is no evidence for glycinergic projections to the MG, so one might suggest that the GABAergic cells are the source of SPN projections to the MG (this would obviously apply to cells with collateral projections to the MG and IC). In fact, in preliminary experiments we have observed SPN cells that were retrogradely labeled from an injection in the MG and were also immunopositive for GAD, a specific marker of GABAergic cells (unpublished results). Future experiments will be needed to assess the prominence of inhibitory projections in the subcollicular projections to the MG.

A majority of MTB cells are principal cells, which have been extensively studied for their association with synaptic calyces of Held and their role in temporal processing and sound localization. The principal cells project to numerous targets including the MSO, LSO, SPN, VNLL and cochlear nucleus (reviewed in Thompson and Schofield, 2000). The MTB also contains stellate and elongate cells (Morest, 1968). Two features of the MTB cells labeled in the present study suggest that the MG-projecting cells are elongate cells. First, elongate cell bodies are smaller and more elongated than those of principal cells; they resemble the MTB cells labeled in the present study (e.g., Figures 8F,G). Second, numerous MTB cells were double-labeled by the dual injections in the MG and the IC. We have shown previously that MTB projections to the IC originate from elongate cells (Schofield, 1994), so collateral projections to the IC and MG must also arise from these cells. It is common to attribute to “the MTB” functions associated with temporal processing, binaural circuitry and sound localization. Such attribution appears appropriate for principal cells, but how much it applies to the other cell types in MTB is not clear. Principal cells do not appear to share any projections with elongate cells; rather, the different projection patterns of elongate cells suggest a different (and yet unknown) function.

Less can be discerned from cells in the other periolivary nuclei, but these areas also reveal correlations between morphology and connections. Small cells in several periolivary nuclei (e.g., the VTB and LTB) project to the MG (present study) and to the IC (Schofield and Cant, 1992), and the present study showed that these cells can be double-labeled by dual injections in IC and MG. Further insights may await advances in our understanding of periolivary functions in general.

### Targets of the projections: parallel pathways and functional implications

As described in the Introduction, physiological and behavioral studies documented auditory function and inputs to the forebrain after lesioning the projections of the IC to the MG. The present results extend our understanding of the extralemniscal pathway that is considered to provide the basis for such function. While the behavioral studies focused on a few types of function (localization, orienting responses, frequency discrimination), it cannot be concluded that these are the only functions served by the extralemniscal system. Support for the idea of multiple functions is provided by a survey of the nuclei of origin: the MSO, LSO, LTB, and MTB are tied to binaural processing and sound localization; the SPN and VLL have been proposed as central players in temporal processing; the VLTg has been associated with the startle reflex and with avoidance/escape behavior, and the paralemniscal area may contribute to pinna movements, and areas medially adjacent to VLL and extending into periolivary regions have been associated with vocalization (e.g., Herbert et al., 1997; Cant and Benson, 2003; Hage et al., 2006; Pollak et al., 2011). Furthermore, although data are quite limited, it appears that multiple neurotransmitters are likely to be involved in these projections. Glutamate and GABA may be involved, suggesting both excitatory and inhibitory effects on MG cells. The paralemniscal area contains neurons that innervate the MGm and appear to release tuberoinfundibular peptide 39, a peptide that presumably plays a neuromodulatory role (Dobolyi et al., 2003). The full complement of neurotransmitters and modulators associated with the subcollicular projections are yet to be determined.

Further insight into function comes from a consideration of the parallel pathways associated with MG subdivisions (Calford and Aitkin, 1983; de Ribaupierre, 1997; Rouiller, 1997). The present results suggest that the MGm is the predominant target of subcollicular auditory projections, but that the dorsal subdivision also receives inputs. The MGm is part of the “polysensory” (or “multimodal”) pathway, reflecting inputs from other sensory systems (particularly the somatosensory system). While early reports suggested that MGm cells were broadly tuned for many stimulus parameters, recent studies show that some MGm cells have narrow tuning similar to that described in the lemniscal pathway and the MGv (Anderson and Linden, 2011). Furthermore, MGm contains neurons that have the shortest latency responses to acoustic stimuli for any MG cells. The MGm thus receives the earliest input about an acoustic stimulus and may provide the forebrain with the earliest information about this stimulus. The present study indicates that the MGm is likely to get information from a very wide range of subcollicular sources. Most or perhaps all of these areas receive direct input from the cochlear nucleus (Warr, 1982; Kandler and Herbert, 1991; Herbert et al., 1997), providing the potential of a single synaptic interruption between the cochlear nucleus and the MG via these extralemniscal projections. The MGm also receives substantial inputs from the IC, including both the central nucleus (the “lemniscal” region) and the lateral cortex (where multimodal cells are located). It remains to be determined whether, and to what extent, the subcollicular and collicular inputs are integrated by MG cells.

A second relevant distinction between the lemniscal and extralemniscal MG subdivisions concerns their projections to higher centers. Lemniscal projections associated with the MGv are the major input to tonotopically-organized core areas of auditory cortex, including primary auditory cortex. Projections from other MG subdivisions make up the extralemniscal system, and differ substantially from the lemniscal pathway. Projections from the MGd and MGsg, often grouped together in the “diffuse” system, terminate in belt areas of cortex, including the temporal auditory field and secondary auditory cortex (AII, as defined in cats). These regions are not tonotopically organized. The MGm, associated with the “polysensory” system, differs from the other divisions by projecting widely to cortex, terminating in most auditory cortical areas. The pattern of axonal termination in the cortical layers differs between the pathways, with lemniscal projections terminating predominantly in layer IV and extralemniscal projections (particularly from MGm) terminating predominantly in layer I (Mitani et al., 1987).

Adrian et al. (1966) lesioned the brachium of the IC bilaterally in cats and showed that acoustically evoked activity was eliminated in some parts of auditory cortex, but not in association and multisensory areas. Such a lesion eliminates all direct projections from the IC to the MG, leaving the projections from subcollicular cells (the ones described in the present report as well as the projections from the cochlear nuclei; Figure 13) as the main sources of auditory input to the thalamus. These results are consistent with the subcollicular pathways activating the extralemniscal MG and then non-primary auditory cortex. Presumably these cortical areas underlie the functions that remain after lesion of the brachium of the IC (described above). In addition, the non-lemniscal MG nuclei are also a major source of projections to the amygdala (LeDoux et al., 1985). These MGm projections have been closely tied to auditory fear conditioning and the role of the amygdala in assessing the behavioral significance of an acoustic stimulus (Weinberger, 2011). The extensive subcollicular projections described in the present study may provide significant input to those circuits that supply the amygdala with auditory information.

### Collateral projections: implications for function and for evolution of neural pathways

We have demonstrated collateral projections to both the MG and the IC from cells in many subcollicular locations. A similar collateral projection characterizes the pathway from the cochlear nucleus to the MG (Schofield et al., 2014). Collateral projections may serve multiple functions. Branching axons could spread the same information to multiple targets, or could perhaps establish a temporal reference (timing point) that could prepare a region for further incoming information about a stimulus. With such roles in mind, it would be particularly interesting to know if individual cells send collateral projections to many targets (i.e., more than the MG and IC). Highly branched axons are characteristic of many cells in the auditory system, such as stellate cells in the cochlear nucleus (reviewed by Cant and Benson, 2003) and principal cells of the MTB (reviewed by Thompson and Schofield, 2000). Such collateral branching could allow a relatively small number of cells, such as those labeled in the present study, to have widespread effects on auditory processing.

Collateral projections may also be viewed from an evolutionary perspective (Noback and Shriver, 1969). Kevetter and Willis (1984) described a scenario for evolution of neural pathways. The ancestral condition is a chain of short links. A second step adds extension of a collateral to a more distant target, while keeping the shorter projection. A subsequent step can be to lose the short-range branch. To apply this scenario to the current study, we use the VLTg as an example. The ancestral condition would be a chain of projections from the cochlear nucleus to the VLTg, then the VLTg to the IC, and finally the IC to the MG (for simplicity, we will ignore laterality here). Some VLTg cells then evolved an additional projection to the MG; these are represented by the double-labeled cells after the dual injections, and include both the large and the small cells in the VLTg. The next evolutionary step was for some cells, namely the large multipolar cells in the VLTg, to lose the collateral branch to the IC, leaving the direct projection from these cells to the MG. It was also readily apparent from the dual injections that the areas studied contain many more IC-projecting cells than MG-projecting cells. This would suggest that the majority of cells reflect the ancestral (short) connections while a small number of cells evolved the longer projections to the MG.

Cell size has been related to both function and evolution in parallel pathways of the visual and somatosensory systems (e.g., Bishop, 1959; Stone, 1983). The extralemniscal auditory pathways have been associated primarily with small cells (e.g., Mitani et al., 1987), and many of the cells labeled in the present study were small compared to unlabeled cells in the same nuclei. The large cells in the VLTg and adjacent regions constitute a notable exception. Similar cells have been described in cats and mustache bats (Henkel, 1983; Casseday et al., 1989; present study, Figure 11E). The large size of these cells may support faster signal transmission, and could underlie some of the short-latency responses in the extralemniscal MG (Anderson and Linden, 2011). The large size of these cells is also reminiscent of Bishop's suggestion that larger cells evolved with the development of longer pathways (Bishop, 1959). These large cells are the same ones discussed in the previous paragraph as lacking collateral projections to the IC. These results suggest that consideration of cell size may provide further insights into both functions and evolution of the subcollicular projections to MG.

## Author contributions

Designed and performed research, analyzed data: all authors; Wrote the paper: Brett R. Schofield.

### Conflict of interest statement

The authors declare that the research was conducted in the absence of any commercial or financial relationships that could be construed as a potential conflict of interest.

## Abbreviations

5n: trigeminal nerve root
7n: facial nerve root
8vn: vestibular nerve root
AQ: cerebral aqueduct
CG: central gray
Cnf: cuneiform nucleus
D: dorsal periolivary nucleus
DCN: dorsal cochlear nucleus
DL: dorsolateral periolivary nucleus
DLL: dorsal nucleus of the lateral lemniscus
FG: FluoroGold
GAD: glutamic acid decarboxylase
GB: green beads
grca: granule cell area of cochlear nucleus
IC: inferior colliculus
icp: inferior cerebellar peduncle
ILL: intermediate nucleus of the lateral lemniscus
lfp: longitudinal fasciculus of the pons
ll: lateral lemniscus
LSO: lateral superior olivary nucleus
LTB: lateral nucleus of the trapezoid body
LVe: lateral vestibular nucleus
Me5: mesencephalic trigeminal nucleus
MG: medial geniculate body
MGd: dorsal division of medial geniculate
MGm: medial division of medial geniculate
MGsg: suprageniculate division of medial geniculate
MGv: ventral division of medial geniculate
MSO: medial superior olivary nucleus
MTB: medial nucleus of the trapezoid body
PL: paralemniscal area
PN: pontine nuclei
PV: posteroventral periolivary nucleus
RB: red beads
s: shell of MG
Sag: sagulum
sp5: spinal trigeminal tract
scp: superior cerebellar peduncle
SPN: superior paraolivary nucleus
SVe: superior vestibular nucleus
VCN: ventral cochlear nucleus
VLL: ventral nucleus of the lateral lemniscus
VLLa: anterior subdivision of VLL
VLLd: dorsal subdivision of VLL
VLLv: ventral subdivision of VLL
VLTg: ventrolateral tegmental nucleus
VTB: ventral nucleus of the trapezoid body.
